# Professional Care Networks of Frail Older People: An Explorative Survey Study from the Patient Perspective

**DOI:** 10.5334/ijic.4721

**Published:** 2020-04-01

**Authors:** Sietske M. Grol, Gerard R. M. Molleman, Michel Wensing, Anne Kuijpers, Joni K. Scholte, Maria T. C. van den Muijsenbergh, Nynke D. Scherpbier, Henk J. Schers

**Affiliations:** 1 Radboud University Medical Center, Radboud Institute for Health Sciences, Department of Primary and Community Care, Nijmegen, NL; 2 Corperate Staff Strategy Development, Radboudumc University Medical Center, Nijmegen, NL; 3 Community Health Service Gelderland-Zuid, Department of Healthy Living, Nijmegen, NL; 4 Department of General Practice and Health Services Research, Heidelberg University Hospital, Heidelberg, DE; 5 Pharos, Centre of Expertise on Health Disparities, Utrecht, NL

**Keywords:** frail elderly, patient perspective, network analysis, network typologies, multidisciplinary teams, primary care

## Abstract

**Background:**

Frail older people living in the community require multidisciplinary care. Despite the fact that patient participation is high on the public agenda, studies into multidisciplinary care mainly focus on the viewpoints of professionals. Little is known about frail older patients’ experiences with care delivered by multidisciplinary teams and their perception of collaboration between professional and informal caregivers.

**Objective:**

To gain more insight into the experiences of frail older patients with integrated multidisciplinary care by mapping the care networks of this patient group and their perception of the interconnection between professional and informal caregivers.

**Methods:**

Survey study to facilitate a care network analysis. Due to the vulnerable health status of the respondents, questionnaires were completed during interviews. Analysis was performed using an iterative process, using both visual and metric techniques.

**Participants:**

44 older persons, considered ‘frail’ by their general practitioner.

**Setting:**

Four general practices in The Netherlands.

**Results:**

The networks of the participants consisted of an average of 15 actors connected by 54 ties. General practitioners were the most common actors in the networks, and were well connected to medical specialists and in-home care providers. The participants did not always perceive a connection between their general practitioner and their informal caregiver. The network analyses resulted in the identification of three subtypes: simple star (n = 16), complex star (n = 16), and sub-group networks (n = 12).

**Conclusions:**

Our findings indicate that the elderly often do not experience the integration of multidisciplinary care as such. This is a real opportunity for MTs to improve their care and to make the patients’ experiences better in line with what they are aiming: allowing patients to live at home as healthy and independently as possible for as long as possible. We showed that informal caregivers often form communication bridges between patients and professionals. Having a better knowledge of the patient perspective enables the gaps in professional care networks of frail older people to be filled and facilitates the anticipation of crisis situations.

## Key points

Patient participation is high on the public agenda, but multidisciplinary teams who offer care to frail older people find it hard to incorporate patients views in their way of working. Our study focused on the care network of the older patient, and more specific on the role of the informal caregiver. In many networks, the informal caregiver was not or only moderately connected with professional actors. Elderly care networks can be rather vulnerable because the power (knowledge, contacts) lies entirely with the patient. Our study suggests that it would be relatively easy to develop materials to enable professionals to map the patient care network, which could then be used as the basis of conversations about the organisation of care, both with the patient and during multidisciplinary team meetings.

## Introduction

### Context

A growing number of frail older people is living in the community [1,2]. Frailty is defined as the accumulation of deficits and diminishing reserves [3]. The complex health needs of this group necessitate a diverse range of healthcare professionals to work together in an integrated, patient-centred way, with the aim to provide good quality end-of-life care [4,5,6,7]. Interprofessional collaboration is crucial for delivering integrated care, preferably involving both healthcare and welfare professionals [8,9]. Moreover, previous studies have shown that it is also very important for professionals to collaborate with informal caregivers [10].

Despite the fact that patient participation is high on the public agenda, studies show that multidisciplinary teams often operate from a professional perspective, and studies into interprofessional collaboration mainly focus on the viewpoints of professionals [11,12,13] or on more quantitative patient outcomes [14,15,16,17]. Furthermore, as previous studies have stressed, increasing communication with informal caregivers is very important in order to actively involve them in the care of older patients with multimorbidities and complex care needs [18,19,20]. Community-based elderly care combines both formal and informal care, with the informal caregivers mainly performing non-skilled care tasks (e.g., administrative/personal care activities) and formal caregivers performing technical and skilled tasks (e.g. nursing care, physiotherapy) [21].

The patient’s perspective is rarely incorporated into the design and execution of multidisciplinary care [22,23,24]. In order to explore ways to structurally involve patient perspectives in the organisation of their care, we were interested in how older people themselves experience their care networks; little is known about frail older patients’ experiences with, and perception of, their multidisciplinary care networks [25,26]. Determining a care network requires the characterisation of an individual’s personal contextual network of healthcare and welfare professionals, and is as such a subjective representation of reality [27]. Previous research into the care networks of frail older people has focused mainly on social networks and social network typologies, and how these relate to health and welfare outcomes [28,29,30,31,32,33]. In the light of integrated multidisciplinary care, recent network research has sometimes targeted a combination of social and professional networks, based on the increasingly accepted idea that formal and informal care for frail older people are inextricably linked [34,35].

### Aim of the study

The aim of the study was to investigate in what way frail older patients describe the network of formal and informal caregivers around them and to establish what they can say about the collaboration between the different actors in the network. This is relevant for the patient-centred organisation of care. Insight in their perspective can help to develop practical tools for the organisation of integrated care for frail older people.

## Methods

### Explorative network analysis study of frail elderly people

Questionnaires were used to map the care networks of 44 frail older people (appendix 1). Due to the frail condition of our participants, we conducted interviews to complete the questionnaires. Ethics approval was not required, according to the Arnhem and Nijmegen Research Ethics Committee (file number 2017–3518). The committee judged that the research participants were not subjected to actions or that no behaviour was imposed on them, that the research had to be classified as an investigation under the Medical Research Act (WMO). Therefore, no positive assessment was required from the Arnhem and Nijmegen Research Ethics Committee or another recognized review committee for its implementation. We followed the criteria for reporting on survey research [36].

### Recruitment of participants

In the Netherlands, all patients are registered with a general practitioner. General practitioners deal with more than 95% of all presented medical problems and arrange referrals to secondary care when required. Dutch general practices provide a comprehensive and patient-oriented approach with a high continuity of care, and can also co-ordinate care for frail older patients with complex care needs [37]. From our network in the Nijmegen area of the Netherlands, we recruited four representative general practices. We ensured heterogeneity between the general practitioner practices using the following criteria: geographical location, population served (deprived, commuter, city, village), years of experience with multidisciplinary elderly care, and scale of the general practice setting.

Potential participants for this study were selected by the general practitioner and/or a practice nurse. We instructed them to select a group of patients in which the following variables were present in a highly varied manner: gender, age, living situation, degree of vulnerability and care needs. To be included, patients had to be labelled ‘frail’ by their general practitioner and discussed in a multidisciplinary team meeting. The criteria for discussion in the multidisciplinary team meeting differed between general practices; two practices screened the whole population of 65+-year-old patients annually using the Easycare-TOS instrument, which includes criteria for defining frailty [38], while the other two practices used case finding. Patients with severe cognitive impairments, for whom the general practitioner estimated that participation in the study would be too burdensome, were excluded. The practice nurse contacted the eligible patients and asked whether the research team could contact them about participating in the study. After obtaining written informed consent, we received their contact information and approached potential participants. We provided them with further details about the interview and made an appointment, and participants received an information sheet to confirm the appointment. As drop-out due to intercurrent illness was foreseen, a larger group then required for theoretical data saturation was approached and also included.

### Interviews and survey questionnaire

The interviews were conducted using a questionnaire, developed by authors A1 (the principal investigator and a health scientist), A6 (a general practitioner and senior researcher), and A4 and A5. Besides data about the professional network, we collected background information. The questionnaire was based on items from existing questionnaires (the TOPICS-MDS [39], Adult and elderly monitor Public Health Service Gelderland-Zuid [40], the Groningen Frailty Indicator [41], the Nijmegen Continuity Questionnaire [42], ‘Polypharmacy in frail elderly: inventory of risks and possible intervention strategies’ [43], the Team Climate Inventory [44] and the Maastricht Social Networking Analysis for people with intellectual disabilities (MSNA-ID) [45]), supplemented with items specific to our study goals. We addressed the following network variables: personal characteristics (e.g. gender, age, marital status), health status (e.g. diseases, medication), persons involved in their care network (professional and informal caregivers), and care co-ordination (‘Is there a care provider who ensures that your care is organised well?’). The survey questionnaire contained more items than reported in this paper (see appendix 1 for further details).

**Figure 1:**
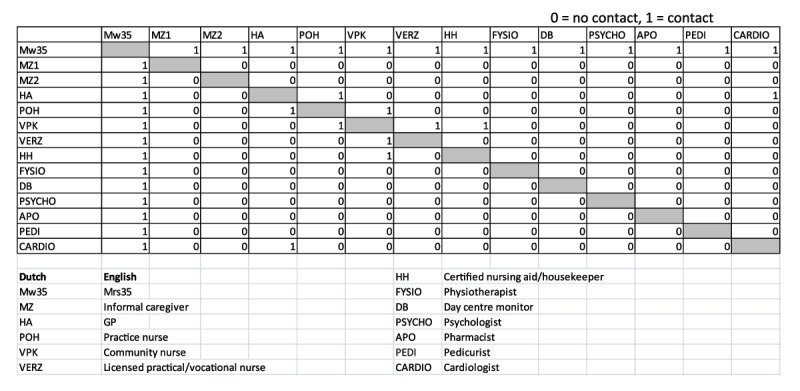
Example of the adjacency matrix for ‘Mrs. 35’.

To collect data about social network variables, we used an adjacency matrix in an A3 format (Figure 1). In four steps, the participants’ networks were portrayed, and connections (ties) among network members (actors) were established. Each actor was assigned both a column and a row in the matrix. The values could be either a ‘0’ (no contact) or a ‘1’ (contact). A ‘1’ meant that actors in the network had mutual contact about the participant, as observed by the participant. This contact could be face to face, over the phone, through letters, or using other means of communication.

The following questions were asked to complete the matrix:

1. Which professional healthcare and welfare professionals were involved in your care in the past 12 months?
2. Who are your most important informal caregivers (max. 3)?
3. With whom does your informal caregiver have contact?
4. Which formal caregivers have contact with each other?

To facilitate the answering of the first question, the interviewer provided a list of the most common care providers in the participant’s community.

The survey questionnaire was tested and piloted. We asked general practitioner colleagues, a welfare worker, and a representative from a regional patient organisation to provide feedback. Subsequently, three frail older people were interviewed as a pilot, which led to further adjustments to the interview guide. The interviews, which lasted 1.5 to 2 hours, took place between June and October 2014 at the home of the participants and were audio-taped. If desired by the participant, an informal caregiver could be present. We ensured anonymity and confidentiality by removing all names from the transcripts and questionnaires, replacing them with consecutive numbers. All respondents received a modest gift as a token of appreciation.

### Analysis

To derive network typologies, we used an iterative process between visual and metrical analysis. The results of the questionnaires were analysed using SPSS version 22. The network data were entered in Microsoft Excel 2007, then transformed into visual networks using NetDraw, a program within the UCInet suite [46]. For each participant, we constructed an ego-network, resulting in network maps with the respondents in the middle. Our iterative process consisted of four phases.

First, authors A1, A2, and A6 performed a visual analysis [47,48] of these care networks and identified typologies based on the number of actors, number of ties, network density, number of sub-groups, and the position of the informal caregivers. For example, in some networks all ties connected only with one or two actors, while in other networks connections were more evenly distributed. This inductive classification led us to define three typologies.

Second, all networks were categorised into one of these three typologies. If there was discussion about the most suitable typology, then we left substantive arguments (‘How does this network work in practice?’) the most important.

Third, we chose network metrics, calculated with UCInet [46], to describe the network typologies and validate the visual analysis (Table 1). These metrics, or structural network indicators, provide quantitative values for specific network characteristics. Five network metrics were used in this study: network size, ties, density, ego-level centrality, and sub-groups. We chose these metrics because with these indicators most networks in our study could be clearly characterized. The first three metrics (network size, ties, and density) are descriptive metrics [49] based on the size of the network and the number of connections it contains. Density is a global measure of interconnectedness within a network. This is important because information can be easily shared in a saturated network [50] and there is a greater chance of mutually shared behaviour and ways of thinking. The remaining two metrics (centrality and sub-groups) are higher-order structural measures: the centrality of the older person in relation to the other actors in the network represents their power (centrality at an ego-level), while the sub-groups (clustering) reveal the extent to which there are sub-networks within the care network [49,51,52].

**Table 1:** Care network metrics of interest and their definitions.

	Metric	Definition

1.	Network size (ego-level)	Number of actors in the network, including the respondent
2.	Ties (network-level)	Number of connections in a network. One tie represents two connections, as within the care networks, all ties are two-way connections
3.	Density (network-level)	Proportion of all possible ties: number of ties/((total number of actors) * (total number of actors – 1))
4.	Centrality (ego-level)	Centrality of the respondent as an attribute of the individual actors, as a consequence of their position
5.	Sub-groups (network-level)	Number of sub-groups. A sub-group is a sub-set of a network in which the actors are more closely and intensely tied to one another than they are to other members of the network

Fourth, authors A1, A2, and A6 combined both the visual analysis and care network metrics to define the network typologies.

## Results

### Participants

Initially, we recruited 65 frail older people from four general practices; however, after the consent conversation, 21 people refrained from participating. Their reasons for not taking part included bad mental and/or physical health, discomfort with having an unknown person in their home, and discomfort about the audio-taping of the interview. We therefore interviewed 44 frail older people, whose characteristics are presented in Table 2. The majority of participants (around 70%) were female, over 80 years of age, faced polypharmacy (≥5 medicines), and had ≥5 chronic disorders. Most of the respondents judged their health to be good to excellent (43%) or reasonable to moderate (43%). According to 50% of our respondents, the general practice co-ordinated their care, often together with in-home care professionals. Almost all respondents (93%) received informal care, which was usually provided by children or other family members.

**Table 2:** Characteristics of the study population (n = 44).

	n (%)

**Gender**	
Male	13 (30)
Female	31 (70)
**Age (average; [min–max]):**	84 [69–98]
65–79	13 (30)
≥80	31 (70)
**Polypharmacy** (≥5 medicines):	32 (73)
**Amount of chronic diseases:**	
2–4	15 (34)
5–7	18 (41)
≥8	11 (25)
Care co-ordinated by*	
general practitioner/practice nurse	12 (50)
In-home care provider	10 (42)
other	2 (5)
no-one	17 (39)
‘I don’t know’	3 (7)
Informal caregiver**:	
spouse	4 (9)
children/son-/-daughter-in-law	32 (73)
other family members	17 (39)
neighbours/friends/acquaintances	15 (34)
none	3 (7)

* Patients could have more than one care co-ordinator. ** Patients could have more than one informal caregiver.

The respondents seemed to be fully capable of identifying the formal and informal caregivers involved in their care network. With help from the interviewer, and the informal caregiver if present, they could identify, on average, 14.5 caregivers in their network (SD 4.2). They were also able to reflect on their interconnectedness (Table 3), although some answers were tentative rather than absolute; for example, respondent:

“Yes, maybe by telephone, but I do not know if they have ever spoken to each other” (patient 70, case 2, female, 82 years old), or respondent.

“I do not know. I got the help I asked for. I would not know whether they co-operate with each other, but I assume that they do.” (patient 24, case 2, female, 86 years old).

**Table 3:** Perceived collaboration between general practices* and others.

Collaboration	General practice* and in-home care providers		General practice and medical specialists		General practice and allied medical** professionals		General practice and social services		General practice and informal caregivers***	

Answer	%	(n****)	%	(n)	%	(n)	%	(n)	%	(n)

Yes	61%	(51)	59%	(61)	29%	(37)	12%	(14)	34%	(60)
No	21%	(17)	20%	(21)	55%	(72)	68%	(78)	59%	(102)
Unknown	13%	(11)	16%	(17)	14%	(18)	19%	(22)	3%	(5)
Presumption	5%	(4)	5%	(5)	2%	(3)	1%	(1)	4%	(7)
*Total*	*100%*	*(83)*	*100%*	*(104)*	*100%*	*(130)*	*100%*	*(115)*	*100%*	*(103)*

* General practices include: general practitioners, practice nurses, practice assistant, pharmacists, and dentists. ** See appendix 2 for a full list of allied medical professionals. *** Respondents reported up to three informal caregivers. **** n = number of answers given, which can transcend the number of respondents.

All but one of the participants mentioned being in contact with their general practitioner in the past 12 months. Pharmacists were present in 95% of the networks, 93% of the respondents reported having at least one informal caregiver, and 91% had a housekeeper. Besides the informal caregiver and the housekeeper, the rest of the ten most common caregivers were involved in healthcare. Professionals from the social domain (appendix 2) were less well represented in the networks. The most commonly mentioned actors from the social domain were representatives from the church (36%), municipal counsellors (25%), and day care monitors (23%).

### Collaboration between caregivers

Because the general practitioner was the most frequently mentioned actor in the networks, we calculated the perceived frequency of collaboration between general practices and several other categories of caregivers (Table 3).

The elderly participants most frequently mentioned collaborations between general practitioners and in-home care professionals, followed by collaborations between general practitioners and medical specialists (e.g. cardiologist, pulmonologist, ophthalmologist). This latter relationship was largely determined by the common respondent response that general practitioners and medical specialists must work together because they send each other letters, respondent:

“Because the cardiologist wrote a whole letter about how things are with me and such. And she copied that and I got it too.” (patient 67, case 1, female, 89 years old).

Table 3 shows that 34% of the respondents reported collaborations between their general practitioner practice and their informal caregivers. The professionals who commonly collaborated with three, four, or five others within a network were pedicurists, housekeepers, and dentists.

### Network typologies

**Figure 2:**
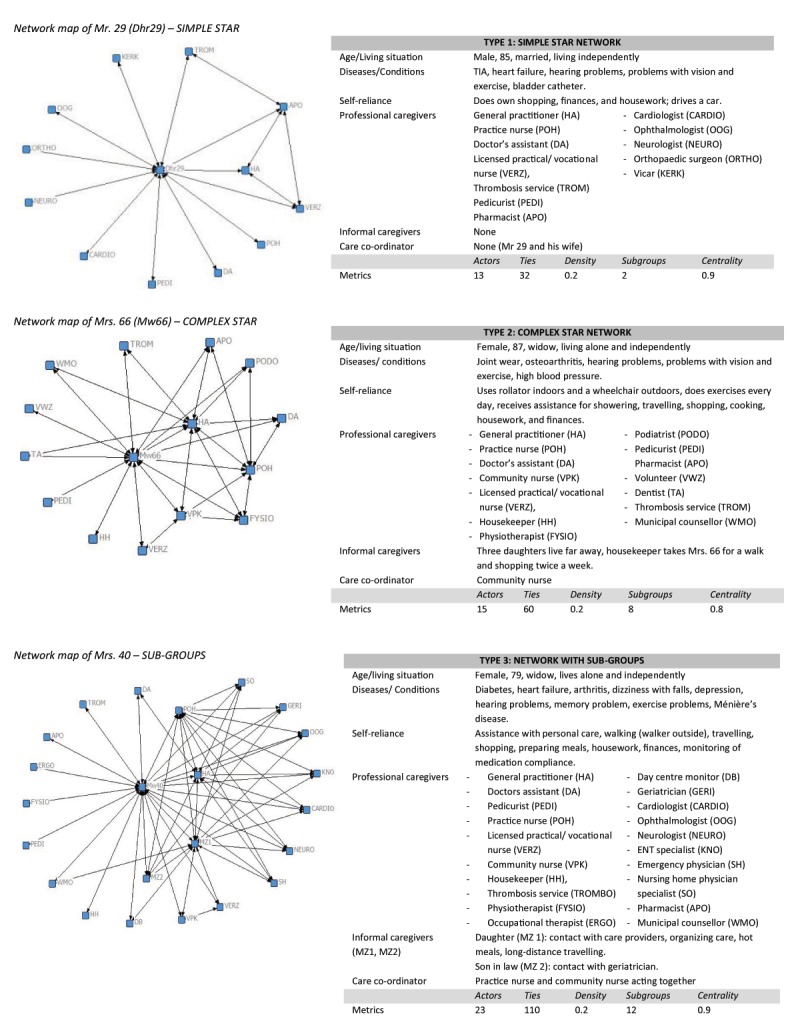
Examples of the three network typologies.

Using the data from the care network matrices, a network map was drawn for each respondent. After four rounds of analyses, we identified three types of networks (Figure 2):

1. Simple star networks with one central actor (the older person) (n = 16),
2. Complex star networks with multiple central actors (n = 16),
3. Networks with >8 sub-groups of actors (n = 12) (A sub-group was defined as a cluster of more than three actors).

The average number of actors in all networks was 14.5.

The average number of ties between the actors was 53.7 (Table 4). Simple star networks had the fewest actors and ties, while the sub-group networks contained, on average, the most actors and ties. The average network density was 0.3 and did not vary much among the types of networks; only the sub-group networks were characterised by a lower average density. The average centrality was 0.8, although in the simple star networks the centrality was higher. The average number of sub-groups was 6.5; the simple star networks had the lowest average number of sub-groups, while the sub-group networks contained the most. Based on these metrics, we concluded that the visual analysis was mainly focussed on differences in the numbers of actors and ties.

**Table 4:** Features of network typologies.

Network type	Number (% of total) Simple star			Number (% of total) Complex star			Number (% of total) Sub-group			Number (% of total) Total		

	16 (36)			16 (36)			12 (27)			44 (100)		

**Characteristic**	**mean**	**min–max**	**SD**	**mean**	**min–max**	**SD**	**mean**	**min–max**	**SD**	**mean**	**min–max**	**SD**

**Number of actors**	11	6–15	3.1	15	12–19	2.2	19	14–26	3.4	14.5	6–26	4.2
**Number of ties**	31.4	14–56	11.9	55.6	38–77	10.5	80.9	56–192	38.2	53.7	14–192	29.3
**Average density**	0.3	0.2–0.6	0.1	0.3	0.2–0.5	0.1	0.2	0.2–0.3	0	0.3	0.2–0.6	0.1
**Centrality**	0.9	0.5–1.0	0.1	0.8	0.6–0.9	0.1	0.8	0.8–0.9	0.1	0.8	0.5–1.0	0.1
**Number of sub-groups**	3.2	0–5	1.4	6.7	6–8	0.8	10.8	9–15	1.7	6.5	0–15	3.3
	**Number (%) of networks of each type in which the informal caregiver is the central person next to the respondent**											

**Informal caregiver**	3 (19)			6 (38)			4 (33)			13 (30)		

In the simple star network, the most ‘power’ (in terms of access to information and contacts) was held by the elderly respondent, while in the complex star networks, at least one additional actor played a central role in the network. This was often an informal caregiver or a primary care professional; therefore, we calculated the centrality of the informal caregiver in each network type. In 30% of the networks, the informal caregiver was the central person alongside the respondent. In the simple star networks, the informal caregiver was least likely to occupy a central position (the informal caregiver occupied a secondary central position in only 19% of the cases), while the informal caregiver occupied the central position alongside the respondent in 38% of complex star type networks.

Sub-group networks consisted of at least nine different sub-groups (groups with > 3 actors), which typically contained, for example, medical specialists, mental healthcare professionals, professionals in dementia care, professionals in primary practice, or allied health professionals. These sub-groups were linked by ‘bridges’, actors in the network that served as connectors between sub-groups.[49] We found that the bridges were often informal caregivers, community nurses, or general practitioners, and that the informal caregivers played a central role alongside the respondent in 33% of sub-group networks. Density in these subgroup networks was lower on average, which can be explained by the higher amount of actors. In Figure 2, examples of the three different network types are presented alongside the background information and metrics of the participant.

## Discussion

### Summary

The mapping of the care network and the relationships between formal and informal caregivers, from the perspective of the frail older patient, has provided new perspectives about patients’ experiences. They are well capable to tell, with assistance and a structured survey, about many aspects of their care network and the way they perceive this. Insight in who collaborates with whom however, is rather tentative. Frail elderly are hardly aware of the organization of multidisciplinary care around them. Coherence in the activities of professionals seems only partially visible by most elderly.

The most common formal and informal caregivers being present in the care networks were general practitioners, pharmacists, informal caregivers, and housekeepers. The position of the informal caregivers in the networks differed; almost 60% of the respondents reported no contact between their informal caregiver and their general practitioner, whilst in most networks both the general practitioner and the informal caregiver formed connections with a larger than average number of actors. The primary care professionals appeared to collaborate mostly with medical specialists and in-home care workers rather than with informal caregivers, social care providers, and professionals from other non-medical domains, who seemed to be at a distance from primary care. This was striking as some of them provide care and services to frail elderly people on a weekly basis (e.g., housekeeper, pedicurist).

In simple star networks, the informal caregivers appeared to be poorly connected or not present at all. This makes this type of network rather vulnerable, as all the ‘power’ (access to information and contacts) lies with the older person. If a crisis occurs in the health status of the older person, the professionals and informal caregivers will have more difficulty connecting with each other to deal with problems that arise. Furthermore, if a professional or informal caregiver fails as an actor and there is no connection with any other caregiver in the network, filling the gap left behind will not be a matter of course. In complex star networks, the informal caregivers were more often present and better connected. In these networks, power was distributed between more actors, making the network less vulnerable. In the sub-group networks, the informal caregivers were well connected and often functioned as ‘bridges’ between different sub-groups. Despite this, the density in these networks remained fairly low on average.

### Comparison with existing literature

Density in the three network types differed. Koetsenruijter [53] emphasised the importance of high densities in care networks, because a more saturated network enables information and knowledge to be more easily shared among actors. A further comparison of the current study with research on social networks by, for instance, Litwin et al. [54] and Cornwall et al. [55] reveals there is still room for improvement; for example, the testing and further development of the three typologies in a larger population would facilitate the statistical underpinning and generalizability of the study. Also, relating the network types to health and welfare indicators would strengthen the conclusions that can be drawn from our study. To the best of our knowledge, no study on professional networks and their relationships with informal caregivers in the care of frail older people has been published. However, in social work and by the nursing and psychiatric profession so called ‘eco-maps’ are used in individual and family counselling [56,57]. An eco-map is a visual display of the informal and formal systems around a patients and serves as a visual representation of support, conflict, or disconnection [58]. Further information on both the professional and social networks could help us to better understand the barriers and facilitators for effective community-based network care [35].

Historically, the relationships between professionals and informal care givers could be characterized as a ‘silent’ hierarchy rather that a collaborative relationship. This was not so much the subject of our research, but it can be an explanation for the fact that the relationships with informal caregivers are not naturally firmly maintained, which can make networks vulnerable [59,60].

As our study showed, informal caregivers can complete the insight into a care network, and can function as bridges to connect different sub-groups of professionals within a network. As Borgatti et al. [61] stated, networks with overlapping sub-groups are considered more stable, and unexpected events can be relatively easily absorbed. Hengelaar et al. [62] suggested that working in collaboration with informal caregivers requires professionals to adopt a different way of functioning. While the focus of the care of vulnerable patient populations is currently often on the patient alone, these results show that specific attention should also be paid to the informal caregiver. This can be difficult to achieve in practice because of the various restrictions experienced by professionals at the policy, legal, and individual levels, as Stephan [63] noted. The initial contact with informal caregivers seems to be particularly challenging, and better strategies are urgently required to facilitate their access to professional support; for example, in-home care organisations could allocate informal caregivers the task of forming a bridge between the patient and their professional care workers, as Jacobs et al. [34] suggests. Wittenberg et al. [64] states that asking informal caregivers for their opinion on the division of responsibilities could make the roles and responsibilities of both informal caregivers and professionals clearer, and improve the collaboration between these actors. Hengelaar et al. [62] stresses that a triad of the older person, the informal caregiver, and a co-ordinating professional is essential. The joint conversation between these three actors is vital to clarify what is important for the older person and what care the informal caregivers and professionals can offer.

### Strengths and limitations

In this study, we performed in-depth interviews with a rather large representative sample of frail older people living in the community in the Netherlands. This provided us with extensive insights into the experiences and views of frail older people in terms of the care and social services they receive, and the interconnectedness they perceived between the professionals and informal caregivers involved in their care. The results of the interviews led to the identification of professional network typologies, which is an innovative way to visualise patient perspectives of community-based elderly care. Our research method was explorative and promising but requires more longitudinal research, and could be linked to patient-based outcome measures and underpinned with statistical calculations to check whether these typologies give the best summary of reality or whether we have overlooked other network typologies.

Although our sample of general practices was heterogeneous, it was rather small. Inclusion of patients through the general practice made it more likely that we would find the general practitioner as a central person. This may have caused selection bias. A different way of inclusion could have given a different outcome. We may have missed frail older people living at home without care through the GP practice, although the chance of this is relatively small. In the Netherlands, all people have a GP who usually has elderly well in view. For example, in 2018, 75+ patients in the Netherlands had an average of 15,5 contacts with their GP practice. [65] The exclusion of patients with severe psycho geriatric problems means that we have missed their experiences. The Dutch setting may mean that these outcomes are representative of the Netherlands, but they might not be generalizable across other countries and cultures; however, our method of mapping care networks could also be used in other countries and populations.

Network metrics allowed us to compare several aspects of the three network typologies; nevertheless, the quantitative measures provided here are for illustrative purposes to demonstrate the types of measures that can be used to analyse these networks. Our data came from a qualitative source, namely the subjective perspective of the respondents, and the results could be enriched by checking the findings with the professionals involved, both in terms of their view of the network and the interconnectedness between the actors involved.

### Implications for practice and research

With this paper, we aimed to deliver usable information to professionals working together in multidisciplinary teams with the ambition to bridge the gap between what older people need and what health care professionals deliver. Our study suggests that frail older people know more, but different things, about their healthcare networks than professionals think. Realizing that the perspective of the patient differs from that the professional, can help MTs to tailor care even better to the needs of the target group. Furthermore, it seems to be advantageous for the co-ordinating professionals to actively connect the actors in care networks because the denser a network, the easier it becomes to share information. We believe it would be relatively easy to develop an instrument to map the network of professionals and informal caregivers caring for a frail older person. Future research should therefore aim to develop materials to enable professionals to map the care network of patients in a simple, rapid manner, which could then be used as the basis of conversations about the organisation of care, both with the older person (and informal caregivers) and during multidisciplinary team meetings. In addition, the three network typologies addressed in this paper could be further underpinned by utilizing them in a broader application linked to health outcomes and enriched with information from co-ordinating care providers and informal caregivers, as was done previously by Perry et al. [35].

## Conclusions

Our explorative study offers insight in the patients’ perspective for collaborating professionals in primary care of the elderly, who share the ambition of further anchoring the perspectives of their patients within the care they supply. Our findings indicate that the elderly often do not experience the integration of multidisciplinary care as such. Elderly care networks can be rather vulnerable because the power (knowledge, contacts) lies entirely with the patient. This is a real opportunity for MTs to improve their care and to make the patient experiences better in line with what they are aiming: allowing patients to live at home as healthy and independently as possible for as long as possible. Older people who did experience that integration were very satisfied with this. They felt secure. We showed that informal caregivers often form communication bridges between patients and professionals. Professionals should be aware of this and invest efforts into developing a relationship with the informal caregivers of frail elderly patients. They can play an important role in experiencing integrated care. Finally, having a better knowledge of the patient perspective, and the possible vulnerability of their network, enables the gaps in these networks to be filled and facilitates the anticipation of crisis situations.

## Exclusive licence statement

“The Corresponding Author has the right to grant on behalf of all authors and does grant on behalf of all authors, a worldwide licence to the Publishers and its licensees in perpetuity, in all forms, formats and media (whether known now or created in the future), to i) publish, reproduce, distribute, display and store the Contribution, ii) translate the Contribution into other languages, create adaptations, reprints, include within collections and create summaries, extracts and/or, abstracts of the Contribution, iii) create any other derivative work(s) based on the Contribution, iv) to exploit all subsidiary rights in the Contribution, v) the inclusion of electronic links from the Contribution to third party material where-ever it may be located; and, vi) licence any third party to do any or all of the above.”

## Patient and public involvement

Members of the elderly panel from ‘netwerk 100’ (a network of care, welfare and educational organisations that develops, together with older people, new projects and products that contribute to improving the well-being of older people in the province of Gelderland, the Netherlands) have contributed to different stages of the study:

– Design of the study;
– Feedback on the interview guide;
– Pilot interviews.

The elderly panel members were not asked about the burden of the interviews, nor were they involved in the in the recruitment to and conduct of the study.

## Transparency declaration

The lead author, Sietske M. Grol, affirms that this manuscript is an honest, accurate, and transparent account of the study being reported; that no important aspects of the study have been omitted; and that any discrepancies from the study as planned (and, if relevant, registered) have been explained.

Sietske M. Grol, the manuscript’s guarantor.

## Data Accessibility Statements

The survey data may be available on reasonable request from the principal investigator (SG). Those data are not publicly available as they contain information that could compromise research participant’s privacy and consent.

## Additional Files

The additional files for this article can be found as follows:

10.5334/ijic.4721.s1 Appendix 1. Interview guide.

10.5334/ijic.4721.s2 Appendix 2. Caregivers in (sub)categories of care and welfare and frequencies of contact in the past 12 months.

10.5334/ijic.4721.s3 Appendix 3. Checklist ‘reporting on survey research’.

## Author Information

Anne Kuijpers died in November 2018.
