# Supplementary material for: Professional Care Networks of Frail Older People: An Explorative Survey Study from the Patient Perspective
Source: Int J Integr Care. 2020 Apr 1;20(1):12. doi: 10.5334/ijic.4721 (PMC7147679; doi:10.5334/ijic.4721)
Supplement: Appendix 1. — Interview guide. [file ijic-20-1-4721-s1.pdf]

Appendix 1. Interview guide

Personal information

1. What is your date of birth?

day — ***month*** — ***year***

2. What is your gender?

- ☐ Male
- ☐ Female

3. What is the highest level of education you have completed?

- ☐ Less than six grades of primary school
- ☐ Six grades of primary school
- ☐ Started but did not complete secondary education
- ☐ Secondary vocational education
- ☐ Secondary school
- ☐ University/higher education

4. What is your marital status?

- ☐ Married
- ☐ Living together
- ☐ Not married/never been married
- ☐ Divorced/living apart
- ☐ Widowed

5. **What is your living situation?**

- ☐ Independently, alone
- ☐ Independently, with other(s)
- ☐ Care home/residential care centre
- ☐ Nursing home

6. **In which country were you born?**

- ☐ The Netherlands
- ☐ Another country: ....

Health

7. **How is your health in general? My health is:**

- ☐ Excellent
- ☐ Good
- ☐ Moderate
- ☐ Bad
- ☐ Very bad

Health (continued)

8. Do you use five or more medications on a daily basis?

- ☐ No
- ☐ Yes

9. Please tick the diseases and conditions that you have or have had in the past 12 months.
You may tick more answers than one.

- ☐ Diabetes
- ☐ Stroke, cerebral haemorrhage, cerebral infarction, or transient ischemic attack
- ☐ Heart failure
- ☐ Cancer
- ☐ Asthma, chronic bronchitis, emphysema, or chronic obstructive pulmonary disease
- ☐ Involuntary urinary incontinence
- ☐ Joint wear (osteoarthritis, wear and tear) of hips or knees
- ☐ Bone decalcification (osteoporosis)
- ☐ Broken hip
- ☐ Fractures other than broken hip
- ☐ Dizziness with falling
- ☐ Prostate complaints due to benign prostate enlargement
- ☐ Depression
- ☐ Anxiety/panic disorder
- ☐ Dementia
- ☐ Hearing problems
- ☐ Problems with seeing
- ☐ Memory problems
- ☐ Problems with movement

Moving

10. How many days a week do you usually move for at least 30 minutes?

- *Only count physical activities that are at least as strenuous as firmly walking or cycling;*
- *Activities shorter than 5 minutes do not count*

	**Rarely or never**	**1**	**2**	**3**	**4**	**5**	**6**	**7**
**Number of active days per week**	☐	☐	☐	☐	☐	☐	☐	☐

Tasks and activities in daily life

11. The following questions are about how you function in everyday life. Please tick the answer that suits you best.

		No	Yes
a.	Do you need help with bathing or showering?	☐	☐
b.	Do you need help getting dressed?	☐	☐
c.	Do you need help when combing your hair or shaving?	☐	☐
d.	Do you need help with going to the toilet?	☐	☐
e.	Do you use incontinence pads?	☐	☐
f.	Do you need help getting out of a chair?	☐	☐
g.	Do you need support when walking?	☐	☐
h.	Do you need help with eating?	☐	☐
i.	Do you need help using the phone?	☐	☐
j.	Do you need help when travelling?	☐	☐
k.	Do you need help shopping?	☐	☐
l.	Do you need help preparing a meal?	☐	☐

**Tasks and activities in daily life (continued)**			
m.	Do you need help with housework?	☐	☐
n.	Do you need help when taking medication?	☐	☐
o.	Do you need help handling money?	☐	☐

Your use of healthcare

12. Have you been admitted to a hospital in the past 12 months?

- ☐ No
- ☐ Yes, one time
- ☐ Yes, two times
- ☐ Yes, three times
- ☐ Yes, more than three times

13. Did you visit your GP's out-of-hours service in the last 12 months or receive a house call from a GP in the night, evening, or weekend?

- ☐ No
- ☐ Yes, one time
- ☐ Yes, two times
- ☐ Yes, three times
- ☐ Yes, more than three times

14. Were you temporarily admitted to a care home/residential care centre in the past 12 months?

- ☐ No
- ☐ Yes, one time
- ☐ Yes, two times
- ☐ Yes, three times
- ☐ Yes, more than three times

Your use of healthcare (continued)

15. Were you temporarily admitted to a nursing home in the past 12 months?

- ☐ No
- ☐ Yes, one time
- ☐ Yes, two times
- ☐ Yes, three times
- ☐ Yes, more than three times

Receiving informal care

Informal care is care you receive from a person close to you (such as your partner, child, neighbour, or friend) when you are ill for a longer period of time, need help, or are disabled. This care can consist of helping with housework, shopping, washing and dressing, keeping you company, transport, arranging money matters, and so on. Informal care is not paid. Your informal caregiver may receive a reimbursement from the municipality. A volunteer who works with a voluntary service is not an informal caregiver.

16. Did you receive informal care in the past 12 months, due to problems concerning your health?

- ☐ Yes, and I still receive informal care
- ☐ Yes, but not anymore →***Proceed to question 19.***
- ☐ No →***Proceed to question 19.***

17. What does this informal care consist of?

You may tick more answers than one.

- ☐ Help with housework (groceries/cleaning)
- ☐ Preparation of hot meals
- ☐ Help with personal care (washing/clothing)
- ☐ Help with medical care
- ☐ Company/comfort/distraction/socialisation
- ☐ Accompaniment and/or transport (such as when visiting the doctor or hairdresser)
- ☐ Money affairs and/or other administration arrangements

Receiving informal care (continued)

18. From whom do you currently receive this care?

You may tick more answers than one.

- ☐ Spouse/partner
- ☐ Children/daughter-in-law/son-in-law
- ☐ Parents (in-law)
- ☐ Other family members
- ☐ Neighbours/friends/acquaintances

Social network and leisure

19. How many times do you have contact with neighbours or people who live in your street?

- ☐ At least once a week
- ☐ Three times per month
- ☐ Two times per month
- ☐ Once per month
- ☐ Less than once per month
- ☐ Seldom or never

Social network and leisure (continued)

20. Some statements now follow. Please indicate the extent to which each statement applies to you and your current lifestyle.

Tick one answer on each line.

		Yes	More or less	No
a.	There is always someone around with whom I can talk about my daily problems.	☐	☐	☐
b.	I miss a really good friend.	☐	☐	☐
c.	I experience a void around me.	☐	☐	☐
d.	I have people on whom I can rely in sad times.	☐	☐	☐
e.	I miss socialising.	☐	☐	☐
f.	I find my circle of acquaintances too limited.	☐	☐	☐
g.	I have many people on whom I can completely rely.	☐	☐	☐
h.	I have enough people with whom I feel closely connected.	☐	☐	☐
i.	I miss having people around me.	☐	☐	☐
j.	I often feel abandoned.	☐	☐	☐
k.	Whenever I need it, I can always contact my friends.	☐	☐	☐

21. Below are a number of activities. Please indicate how often you practice these activities.
Tick one answer on each line.

		(Almost) daily	Weekly	A few times a month	Once a month or less	Hardly ever or never
a.	Visiting / receiving visitors	☐	☐	☐	☐	☐
b.	Keeping in contact over the phone or internet (for instance, via e-mail or Skype)	☐	☐	☐	☐	☐
c.	Taking part in an association (for instance, going to a club)	☐	☐	☐	☐	☐

Support and services

22. Have you ever heard of [name of the local information desk for care and welfare] and/or [name local organisation for wellbeing of the elderly]? *You may tick more answers than one.*

- ☐ No →Go to question 25.
- ☐ Yes, via an advisor for the elderly
- ☐ Yes, via another caregiver
- ☐ Yes, via family/friends/acquaintances/neighbours
- ☐ Yes, via an information letter
- ☐ Yes, via a leaflet
- ☐ Yes, via the internet (e.g., website of [name local organisation for wellbeing of the elderly])
- ☐ Yes, via articles in the local newspaper [name of the local newspaper]
- ☐ Yes, via other means

23. [Name of the local information desk for care and welfare**] and/or [**name local organisation for wellbeing of the elderly**] provide support and organise activities. The following are opportunities for support and activities. Please indicate whether you are aware of these and, if so, whether you have made use of any of these possibilities.**
Tick one answer on each line.

		No	Yes, I am aware, but I do not use it	Yes, I am aware and I make (previously made) use of it
a.	Support for older people from a social care service provider [*name of local caregiver*]	☐	☐	☐
b.	Individual transport [*name of local elderly transport*]	☐	☐	☐
c.	Days out for elderly people	☐	☐	☐
d.	Help with small jobs in home or garden, accompaniment to hospital visits, groceries	☐	☐	☐
e.	Help with finance	☐	☐	☐
f.	Information about preventing falls	☐	☐	☐
g.	Meal service or eating together	☐	☐	☐
h.	Movement activity ("More Exercise for the Elderly") (e.g., gym, dance, walking, swimming)	☐	☐	☐
i.	Club (e.g., billiards, cards, knitting, choir)	☐	☐	☐

		No	Yes, I am aware, but I <u>do not</u> use it	Yes, I am aware and I make (previously made) use of it
j.	Volunteer who takes part in activities with you (e.g., games, trips, shopping)	☐	☐	☐
k.	A course	☐	☐	☐
l.	Other support / activities	☐	☐	☐

24. **Has anyone from** [name of the local information desk for care and welfare] **and/or** [name local organisation for wellbeing of the elderly] **visited you?**

- ☐ No
- ☐ Yes

Caregivers

25. **Which caregivers have you had contact with in the last 12 months?**
<u>Caregivers:</u> people who give care and are paid for it, or those who give care voluntarily in association with an organisation.
<u>Having contact:</u> visit to your caregiver, house call from your caregiver, a conversation over the phone with your caregiver. This <u>does not mean</u> calling for a repeat prescription.
You may tick more answers than one.

- ☐ GP
- ☐ Practice nurse
- ☐ GP assistant
- ☐ Community nurse
- ☐ Licensed practical/vocational nurse
- ☐ Certified nurse aid/housekeeper
- ☐ Physiotherapist
- ☐ Occupational therapist
- ☐ Dietician
- ☐ Provider of social care service for older people
- ☐ Day centre monitor
- ☐ Mental health nurse in primary practice /mental health nurse
- ☐ Psychologist
- ☐ Social worker
- ☐ Speech therapist
- ☐ Chiropodist/podiatrist
- ☐ Cesar remedial therapist
- ☐ Mensendieck remedial therapist
- ☐ Social service community team
- ☐ Pharmacist
- ☐ Dentist/dental hygienist
- ☐ Pedicurist
- ☐ Thrombosis service provider

Caregivers (continued)

- ☐ Municipal counsellor
- ☐ Priest / pastor / others from church
- ☐ Volunteer from an organisation (e.g., [*local volunteer organisation*])
- ☐ Alternative healer (e.g., homeopath)
- ☐ Another caregiver: …………………………………………………………………………….

26. Which doctors from the hospital have you had contact with in the past 12 months?
You may tick more answers than one.

- ☐ Does not apply
- ☐ Cardiologist
- ☐ Ear, nose, and throat specialist
- ☐ Pulmonologist
- ☐ Gynaecologist
- ☐ Neurologist
- ☐ Ophthalmologist
- ☐ Rehabilitation specialist
- ☐ Dermatologist
- ☐ Internist
- ☐ Geriatrician
- ☐ Nursing home physician specialist
- ☐ Emergency physician
- ☐ Gastrointestinal and liver specialist
- ☐ Orthopaedist/orthopaedic surgeon
- ☐ Psychiatrist
- ☐ Rheumatologist
- ☐ Urologist
- ☐ Radiologist/radiotherapist
- ☐ Oncologist
- ☐ Other medical specialist: ....

27. Is there a care provider who ensures that your care is organised well?

- ☐ No → ***Go to question 29.***
- ☐ Yes

28. Which of your care provider(s) takes care of this?

You may tick more answers than one.

- ☐ GP
- ☐ Practice nurse
- ☐ Community nurse
- ☐ Nursing home physician specialist

Caregivers (continued)

29. The following questions are about the collaboration between your care providers.
Tick one answer on each line.

		Strongly agree	Agree	Neutral	Disagree	Strongly disagree	Don't know
a.	My caregivers transfer information to each other very well.	☐	☐	☐	☐	☐	☐
b.	My caregivers collaborate very well.	☐	☐	☐	☐	☐	☐
c.	Care provided by these caregivers is very well connected.	☐	☐	☐	☐	☐	☐
d.	My caregivers always know what each other is doing.	☐	☐	☐	☐	☐	☐
e.	My caregivers work together to develop and apply new ideas.	☐	☐	☐	☐	☐	☐
f.	My caregivers always interact with each other in a pleasant way.	☐	☐	☐	☐	☐	☐

Space for comments:

End of the questionnaire. Thank you for your co-operation!
