# Supplementary material for: Professional Care Networks of Frail Older People: An Explorative Survey Study from the Patient Perspective
Source: Int J Integr Care. 2020 Apr 1;20(1):12. doi: 10.5334/ijic.4721 (PMC7147679; doi:10.5334/ijic.4721)
Supplement: Appendix 2. — Caregivers in (sub)categories of care and welfare and frequencies of contact in the past 12 months. [file ijic-20-1-4721-s2.pdf]

Appendix 2. Caregivers in (sub)categories of care and welfare and frequencies of contact in the past 12 months

	Subcategories healthcare		Abbr.	Caregivers	Frequency of contact in past 12 months % / n	
HEALTH CARE	1. Providers of primary care, medical care	1.1	HA	General practitioner	93%	41
		1.2	POH	Practice nurse	68%	30
		1.3	DA	GP assistant	66%	29
		1.4	APO	Pharmacist	96%	42
		1.5	TA	Dentist	30%	13
	2. Providers of primary care, in-home care	2.1	VPK	Community nurse	48%	21
		2.2	VERZ	Licensed practical/vocational nurse	66%	29
	3. Providers of primary care, other medical care	3.1	VPKS	Specialist nurse	0%	0
		3.2	ALT	Alternative healer	7%	3
		3.3	VPKO	Other nurse	5%	2
	4. Allied health professionals	4.1	FYSIO	Physiotherapist	55%	24
		4.2	ERGO	Occupational therapist	18%	8
		4.3	CESAR	Cesar remedial therapy	5%	2
		4.4	MD	Mensendieck remedial therapy	2%	1
		4.5	DIET	Dietician	21%	9
		4.6	LOGO	Speech therapist	2%	1
		4.7	PODO	Chiropodist/podiatrist	14%	6
		4.8	PEDI	Pedicurist	73%	32
		4.9	MH	Dental hygienist	0%	0
		4.10	OHP	Other provider of allied care	11%	5
	5. Providers of medical specialist care	5.1	TROM	Thrombosis service provider	27%	12
		5.2	MS	Medical specialist	75%	33
		5.3	VPKZ	Hospital nurse	7%	3
		5.4	OHZ	Other hospital-based specialist care provider	2%	1
	6. Providers of mental care	6.1	POHGGZ	Mental health nurse primary care	2%	1
		6.2	SPV	Mental health nurse	21%	9
		6.3	PSYCHO	Psychologist	9%	4
		6.4	ZTBD	Dementia counsellor	2%	1
		6.5	OHPS	Provider of other mental healthcare	2%	1
SOCIAL CARE	Subcategories social care			Providers of social care		
	6. Professional providers of social care	7.1	HH	Certified nursing aid/housekeeper	89%	39
		7.2	OA	Provider of social care service for older people	14%	6
		7.3	DB	Day centre monitor	23%	10
		7.4	MW	Social worker	7%	3
		7.5	WMO	Municipal counsellor	25%	11
		7.6	KERK	Priest/pastor/others from the church	36%	16
		7.7	OHW	Provider of other social care	9%	4
	8. Voluntarily providers of social care	8.1	VWZ	Volunteer from Zonnebloem	25%	11
		8.2	VW	Volunteer social care service for older people	14%	6
		8.3	OVW	Other volunteer	5%	2
INFOR MAL	9. Informal caregivers	9.1	MZ	Informal caregiver	91%	68

Appendix 2. (continued) Caregivers in (sub)categories of care and welfare and frequencies of contact in the past 12 months

Specification Medical Specialists

**Abbreviation**	**Caregiver**
CARDIO	Cardiologist
KNO	Ear, nose, and throat specialist
LONG	Pulmonologist
GYN	Gynaecologist
NEURO	Neurologist
OOG	Ophthalmologist
REVI	Rehabilitation specialist
DERMA	Dermatologist
INTER	Internist
GERI	Geriatrician
SO	Nursing home physician specialists
SH	Emergency physician
MDL	Gastrointestinal and liver specialist
ORTHO	Orthopaedist/orthopaedic surgeon
PSYCHI	Psychiatrist
REUMA	Rheumatologist
URO	Urologist
RADIO	Radiologist/radiotherapist
ONCO	Oncologist
MSO	Other medical specialist
