# Supplementary material for: Professional Care Networks of Frail Older People: An Explorative Survey Study from the Patient Perspective
Source: Int J Integr Care. 2020 Apr 1;20(1):12. doi: 10.5334/ijic.4721 (PMC7147679; doi:10.5334/ijic.4721)
Supplement: Appendix 3. — Checklist ‘reporting on survey research’. [file ijic-20-1-4721-s3.pdf]

Appendix 3 Checklist 'reporting on survey research' (Kelley et al. 2003)[32]

Key point	*Page number*
(1) Explain the purpose or aim of the research, with the explicit identification of the research question.	2
(2) Explain why the research was necessary and place the study in context, drawing upon previous work in relevant fields (the literature review).	2
(3) Describe in (proportionate) detail how the research was done.<br>(a) State the chosen research method or methods, and justify why this method was chosen.<br>(b) Describe the research tool. If an existing tool is used, briefly state its psychometric properties and provide references to the original development work. If a new tool is used, you should include an entire section describing the steps undertaken to develop and test the tool, including results of psychometric testing.<br>(c) Describe how the sample was selected and how data were collected, including:<br>(i) How were potential subjects identified?<br>(ii) How many and what type of attempts were made to contact subjects?<br>(iii) Who approached potential subjects?<br>(iv) Where were potential subjects approached?<br>(v) How was informed consent obtained?<br>(vi) How many agreed to participate?<br>(vii) How did those who agreed differ from those who did not agree?<br>(viii) What was the response rate?	2-4
(4) Describe and justify the methods and tests used for data analysis.	3-4
(5) Present the results of the research. The results section should be clear, factual, and concise.	4-7
(6) Interpret and discuss the findings. This 'discussion' section should not simply reiterate results; it should provide the author's critical reflection upon both the results and the processes of data collection. The discussion should assess how well the study met the research question, should describe the problems encountered in the research, and should honestly judge the limitations of the work.	8-9
(7) Present conclusions and recommendations.	9-10
